# Depression and anxiety during the first and second waves of the COVID‐19 pandemic in two large, prospective, aging cohorts in rural and urban India

**DOI:** 10.1002/hsr2.901

**Published:** 2022-11-08

**Authors:** Jonas S. Sundarakumar, Abhishek L. Menesgere, Shafeeq K. S. Hameed, SANSCOG & TLSA Study Teams, Vijayalakshmi Ravindranath

**Affiliations:** ^1^ Centre for Brain Research Indian Institute of Science Bangalore India

**Keywords:** aging adults, anxiety, COVID‐19 pandemic, depression, India

## Abstract

**Introduction:**

The COVID‐19 pandemic resulted in a wide variety of adverse consequences, including disruption of long‐term, human research studies globally. Two long‐term, prospective, aging cohort studies, namely, Srinivaspura Aging, Neurosenescence and COGnition (SANSCOG) study and Tata Longitudinal Study of Aging (TLSA), conducted in rural and urban India, respectively, had to be suspended during first and second waves of COVID‐19.

**Methods:**

We conducted telephonic assessments to screen for depression and anxiety in the above two cohorts comprising of adults ≥45 years, during the first wave (2020) and second wave (2021) lockdown periods in India. Further, we included depression assessments data from two additional time periods—pre‐COVID (2019) and the “inter‐wave” period (between the first and second waves) to compare proportions of depression in these cohorts, during four distinct time periods—(i) pre‐COVID, (ii) COVID first wave lockdown, (iii) inter‐wave period, and (iv) COVID second wave lockdown (rural: 684, 733, 458, 611 and urban: 317, 297, 204, 305 respectively).

**Results:**

During COVID first wave, 28.8% and 5.5% had depression and anxiety, respectively in the rural cohort. Corresponding figures in the urban cohort were 6.5% and 1.7%. During second wave, 28.8% of rural subjects had depression and 3.9% had anxiety, whereas corresponding figures in urban subjects were 13.1% and 0.66%. During the above‐mentioned four time periods, proportions of depression were: rural—8.3%, 28.8%, 16.6%, 28.8%; urban—12%, 6.1%, 8.8%, 13.1%.

**Conclusions:**

Multi‐fold increase in depression among aging, rural Indians during first and second waves, with high depression among subjects ≥65 years and those with comorbidities during the first wave, is concerning. Urgent public health measures are needed to address this added mental health burden and thereby, prevent further potential adverse consequences.

## 1 INTRODUCTION

The COVID‐19 pandemic resulted in an enormous adverse impact on humankind and caused tremendous suffering all over the world. India was substantially impacted by both the first and second waves of the pandemic in 2020 and 2021, respectively. The first wave of the pandemic resulted in stringent nationwide lockdowns from late March 2020 till the end of May 2020.^1^ Further, phased easing of restrictions continued for several months after this. Just as the country was on the road to recovery from the first wave, the second wave hit in April 2021. The impact of the second wave was far more detrimental than the first wave, not only because of the higher magnitude of cases and deaths but also due to its precipitous onset.^2^, ^3^, ^4^

The dimensions of the adverse impact of this pandemic have extended far beyond the direct or acute effects of the infection or disease manifestations. In particular, the psychological impact has been prominent, though understudied and not well understood. There could be several possible reasons for substantial, adverse mental health consequences due to COVID‐19 in developing countries such as India. These include widespread fear^5^, ^6^ and stigma^7^, ^8^ regarding the disease, disruption in accessing healthcare during the lockdowns,^9^, ^10^ economic impact,^11^ overburdened, and disparate healthcare systems including weak primary healthcare infrastructure^12^, ^13^ and the already high burden of mental health disorders in the country.^14^, ^15^ In this scenario, it is crucial to understand the extent and nature of the psychological impact of COVID‐19 in India, so that appropriate preventive or mitigative interventions can be developed to counter or mitigate the adverse consequences.

Older adults are not only more vulnerable to COVID‐19 infection along with poorer disease outcomes,^16^ they are likely to have suffered considerably due to the psychosocial and economic impact of the pandemic and its associated restrictions.^17^, ^18^, ^19^, ^20^, ^21^ Though studies^22^, ^23^, ^24^, ^25^, ^26^, ^27^, ^28^, ^29^ from other parts of the world have attempted to study the psychological impact of the pandemic in various population groups, such studies from India are limited,^30^, ^31^, ^32^, ^33^, ^34^ particularly in the aging population.^35^, ^36^, ^37^, ^38^

We are conducting two large‐scale longitudinal cohort studies—Srinivaspura Aging, Neurosenescence and COGnition (SANSCOG) study in rural India (projected *n* = 10,000) since 2018 and Tata Longitudinal Study of Aging (TLSA) in urban India (projected *n* = 1000) since 2015. These studies are aimed at studying the diverse cognitive trajectories of aging and thereby, identifying risk and protective factors for aging‐related, neurodegenerative disorders such as dementia. Both these studies had to be halted during the first and second wave COVID‐19 lockdowns in India. However, during both these lockdown periods, we conducted telephonic assessments on consenting subjects from both cohorts to screen for depression and anxiety. In this paper, we present the proportions of depression and anxiety in both the above cohorts during the first and second waves of the pandemic. Further, we utilized data from depression assessments that were carried out as part of the periodically scheduled clinical assessments in these two cohorts to establish two additional COVID‐related time periods—“pre‐COVID period” (assessments done in 2019) and “inter‐wave period” (assessments done between the first wave and second waves, when the cohort studies had briefly resumed). With this additional data, we compared proportions of depression in the rural and urban cohorts, during four distinct time periods—(i) pre‐COVID period, (ii) first‐wave lockdown period, (iii) inter‐wave period, and (iv) second‐wave lockdown period. We hypothesized that there would be significant rise in the proportion of depression during the first wave and second wave of the pandemic as compared with either the pre‐COVID or the inter‐wave periods in subjects from both cohorts.

## 2 METHODS

### 2.1 Study design

Mixed methods study with cross‐sectional assessments as well as utilizing previously acquired longitudinal data for comparison.

### 2.2 Setting

Community setting in the villages of Srinivaspura (SANSCOG study) and urban Bangalore (TLSA). Both study sites are approximately 60 miles apart, located in the southern Indian state of Karnataka.

### 2.3 Cohort characteristics

Rural (SANSCOG) cohort participants are village‐dwelling, predominantly from a low/lower‐middle income (annual household income <200,000 Indian Rupees) agricultural background, with limited levels of literacy (ability to both read and write with understanding, in any language) and have not been exposed to substantial lifestyle changes or modernization. However, the urban (TLSA) cohort subjects are city‐dwelling, highly educated, belong to the working middle/high‐income (annual household income >200,000 Indian Rupees), and have seen significant lifestyle changes due to urban living in the last few decades. Details of the two cohorts, sampling frames, and recruitment strategies are given in Appendix I.

### 2.4 Study sample

The study sample includes cognitively healthy, aging individuals (45 years and above), who were enrolled in two, prospective, aging cohorts, namely, SANSCOG and TLSA (described earlier). In the rural cohort, 733 and 611 subjects consented and underwent telephonic assessments for depression and anxiety during the first wave (June 22–August 27, 2020) and second wave (May 7–May 31, 2021), respectively. Similarly, in the urban cohort, 297 and 305 subjects consented and underwent these assessments during the first wave (June 1–June 16, 2020) and second wave (May 31–June 8, 2021), respectively.

### 2.5 Ethics and informed consent

Both SANSCOG and TLSA studies have been cleared by the Institutional Ethics Committee of the Centre for Brain Research, Indian Institute of Science, and the collaborating institutions. Written, informed consent was obtained from all participants before recruitment into the two studies. During the lockdown periods of the first and second waves of the COVID‐19 pandemic, we initially attempted to telephonically contact all participants in both rural (SANSCOG) and urban (TLSA) cohorts to enquire about their well‐being and to provide them awareness regarding COVID‐19‐related safety precautions as well as medical guidance, when necessary. Further details of these initial rounds of calls have been presented in another manuscript.^39^ During these calls, participants from both cohorts were informed about the telephonic assessments for depression and anxiety and willing participants provided voluntary, telephonic consent for these assessments.

### 2.6 Assessments

To avoid bias, we used the same assessment instruments in both cohorts during both waves of the pandemic. Depression was assessed by using the 7‐item version of the Geriatric Depression Scale (GDS‐7) and anxiety symptoms were assessed using the 7‐item version of the Generalized Anxiety Disorder Questionnaire (GAD‐7). Details of the scales, administration, scoring, and interpretation are given in Appendix II.

**Figure 1:**
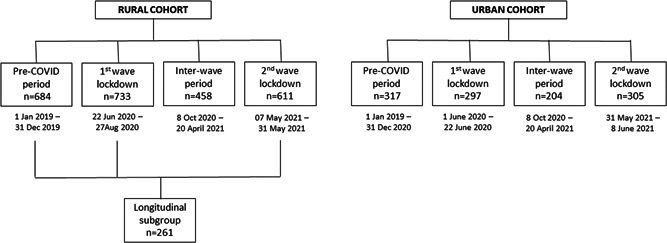
The flow diagram represents the number of rural (SANSCOG) and urban (TLSA) cohort participants at different stages of recruitment for undergoing telephonic assessments for depression and anxiety during the first and second wave lockdowns periods of COVID‐19 in India. Also depicted below are number of rural (SANSCOG) and urban (TLSA) cohort participants for whom depression assessment data were available during four distinct COVID‐related time periods: (i) Pre‐COVID period, (ii) COVID first wave lockdown period, (iii) COVID inter‐wave period, and (iv) COVID second wave lockdown period. SANSCOG, Srinivaspura Aging, Neurosenescence and COGnition; TLSA, Tata Longitudinal Study of Aging.

Even before the onset of the pandemic, regular, in‐person, clinical assessments of both our cohorts included depression assessments (GDS‐30) as well as collecting information on self‐reported history of depression, alcohol use, smoking, and medical comorbidities. Similarly, between the first and second wave lockdowns, when we briefly resumed both studies (October 8, 2020–April 21, 2021), all the above‐mentioned information was collected during the clinical assessments done in‐person (as part of the regular SANSCOG & TLSA study schedules). Thus, we had two more time periods, during which depression assessments were performed in these cohorts—one, before the onset of the COVID‐19 pandemic (referred to in this paper as “pre‐COVID” period) and another, when assessments were conducted after the first wave and until the onset of the second wave (referred to in this paper as “inter‐wave” period). The four distinct time periods when depression assessments were carried out in the rural and urban cohorts are represented in Figure 1. Proportions of subjects screened to have depression during the above four time periods were compared in both the cohorts.

Further, in the rural cohort, we identified a longitudinal subgroup of subjects (*n* = 261), who had GDS data for pre‐COVID, COVID first‐wave, and second‐wave lockdown periods. This enabled us to observe how the proportion of depression differed in the same subgroup of subjects during these three distinct time periods. We could not include the inter‐wave period in this analysis due to the parent studies' follow‐up time frames (once in 3 years for subjects <65 years and once in 2 years for subjects ≥65 years). We analyzed how factors such as age, gender, literacy, presence of co‐morbidities, past history of depression, alcohol use, and tobacco use played out during these different time periods—before the pandemic and during the first and second wave lockdown periods.

### 2.7 Statistical analysis

Data was entered in excel sheets and were subsequently exported to the JASP open‐source software (version 0.14.1). Differences in overall proportions of depression during different assessment periods in the rural as well as urban cohort were analyzed. Further analysis of the longitudinal subgroup of SANCOG cohort subjects based on age, gender, presence of comorbidity, past of depression, and substance use was done using Pearson's *χ*² test. A *p*‐value of <0.05 was considered statistically significant. Cramér's *V* was also reported wherever the *p*‐value was significant and interpreted as the effect size measure (Cramér's *V* <0.2—weakly associated, 0.2–0.6—moderately associated, and >0.6—strongly associated). There was no missing data for depression and anxiety assessments in both the cohorts and hence, no imputation analysis was done.

## 3 RESULTS

The flow diagram depicting number of rural and urban cohort participants who underwent telephonic assessments for depression and anxiety during the first‐ and second‐wave lockdowns are displayed in Figure 1. Mean age, gender, and literacy status of rural and urban participants belonging to the 4 studied groups (pre‐COVID, COVID first wave lockdown period, COVID inter‐wave period, and COVID second wave lockdown period) are presented in Table 1. Among the longitudinal subgroup participants in the rural cohort (*n* = 261), who had depression assessments in the pre‐COVID as well as COVID first wave and second wave, 56% (147/261) were males, mean age was 55.6 ± 8.8 years (males: 55.9 ± 8.9, females: 55.2 ± 8.6) and literacy rate was 70%; 183/261 (males: 129/147;88%, females: 54/114; 47%).

**Table 1:** Sociodemographic characteristics of rural and urban participants during the four studied time periods

Rural cohort	Pre‐COVID period	COVID first‐wave lockdown period	COVID interwave/unlock period	COVID second‐wave lockdown period	*p*‐value
No. of participants	684	733	458	611	‐
Mean age ± SD (years)	58.8 ± 10.3	58 ± 9.5	60.6 ± 10.1	58.4 ± 9	<0.001^b^ *η*² = 0.009
Gender (males)	327/680^a^ (48%)	366/728^a^ (50%)	241/458 (53%)	330/611 (54%)	0.16
Literacy status (literate)	369/677^a^ (54.5%)	466/726^a^ (64.2%)	286/458 (62.4%)	436/610^a^ (71.5%)	<0.001 Cramér's *V* = 0.12
Income class (low/lower‐middle class)	635/677^a^ (93.8%)	655/726^a^ (90.2%)	302/448^a^ (67.4%)	451/610^a^ (73.9%)	<0.001 Cramér's *V* = 0.28

**Urban cohort**	**Pre‐COVID period**	**COVID first‐wave lockdown period**	**COVID interwave/unlock period**	**COVID second‐wave lockdown period**	***p*‐value**
No. of participants	317	297	204	305	‐
Mean age ± SD (years)	66.7 ± 9.1	66.8 ± 9.1	65.6 ± 8.9	65.8 ± 9	0.989
Gender (males)	178/317 (56.2%)	172/297 (57.9%)	123/204 (60.3%)	171/305 (56.1%)	0.764
Literacy status (literate)	317/317 (100%)	296/297 (99.7%)	204/204 (100%)	305/305 (100%)	0.426

*Note*: All participants in the urban cohort belonged to upper‐middle/high‐class income group.

^a^ Missing sociodemographic data.

^b^ Post hoc test revealed that only the mean age of COVID interim/unlock period group was significantly different from all the other groups.

**Figure 2:**
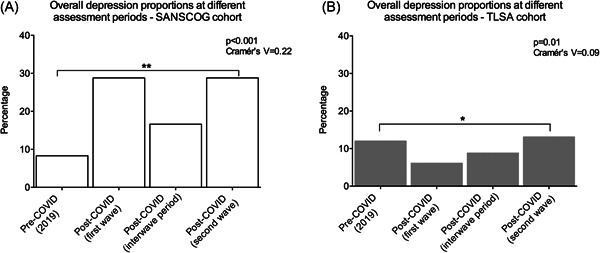
This figure depicts the different proportions in depression in the rural and urban cohort subjects four distinct COVID‐related time periods: (i) Pre‐COVID period (*n* = 684 rural and 317 urban), (ii) COVID first wave lockdown period (*n* = 733 rural and 297 urban), (iii) COVID inter‐wave period (*n* = 458 rural and 204 urban), and (iv) COVID second wave lockdown period (*n* = 611 rural and 305 urban).

The proportion of subjects with depression in the rural cohort during the first and second waves of the COVID‐19 pandemic was 28.8% (211/733) and 28.8% (176/611), respectively. Similarly, 5.5% (40/733) and 3.9% (24/611) were screened to have anxiety during the first and second waves of the pandemic, respectively. On the other hand, in the urban cohort, the proportions of subjects with depression were 6.1% (18/297) and 13.1% (40/305) during the first and second waves, respectively. The corresponding numbers for subjects screened to have anxiety were 1.7% (5/297) and 0.66% (2/305). In addition, the proportions of depression during the pre‐COVID period were 8.3% (57/684) for rural and 12% (38/317) for urban subjects. Similarly, proportions of depression during the COVID inter‐wave period were 16.6% (76/458) for rural and 8.8% (18/204) for urban subjects. Thus, we found varying proportions in depression between the rural and urban cohorts during the above‐mentioned four time periods (Figure 2).

**Figure 3:**
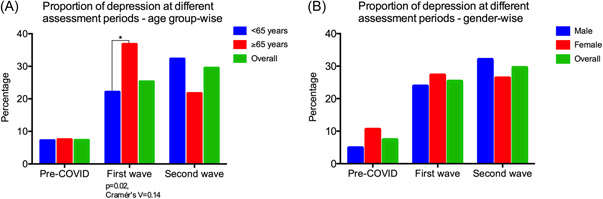
This figure shows the changes in proportions of depression according to age group and gender among a longitudinal subgroup of rural cohort subjects (*n* = 261), who had depression assessments pre‐COVID as well as during the first wave and second waves of COVID.

Further, among the longitudinal subgroup of rural subjects (*n* = 261), who had undergone GDS assessments during pre‐COVID, COVID first‐wave, and second‐wave lockdown period, we found that the proportion of subjects screened to have depression differed significantly (*p* < 0.001) between the above three time periods: 7.3%; 19/261 (pre‐COVID), 25.3%; 66/261 (COVID first wave), and 29.5%; 77/261 (COVID second wave). On age‐stratifying the above longitudinal subgroup (<65 years and ≥65 years), we observed that older adults ≥65 years had significantly higher proportion of depression than those <65 years (37.8%; 20/53 vs. 22.1%; 46/208, *p* = 0.02) during the first wave of the pandemic. However, no significant difference between these two age groups was observed in the pre‐COVID and COVID second‐wave proportions of depression (Figure 3). On gender‐stratifying these subjects, we observed a female preponderance in the pre‐COVID proportion of depression (10.5%; 12/114 females vs. 4.8%; 7/147 males), though this was not statistically significant (*p* = 0.07). No significant gender difference was observed in the proportions of depression during the first or second wave of the pandemic (Figure 3). On stratifying these subjects by literacy (literate vs. illiterate) we did not observe any significant difference in the proportions of depression between the two groups during any of the three time periods. Similarly, there was no significant difference in depression between those with alcohol or tobacco use (self‐reported) and those without, as depicted in Table 2.

**Table 2:** Comparison of proportions of depression between groups stratified by literacy status, tobacco use, and alcohol use

Assessment period	Literacy status		*χ* ^2^, *p*	Tobacco use		*χ* ^2^, *p*	Alcohol use		*χ* ^2^, *p*
	Illiterate	Literate		Yes	No		Yes	No	
Pre‐COVID	5/78 (6.4%)	14/183 (7.7%)	*χ* ^2^ = 0.12, *p* = 0.72	9/75 (12%)	10/186 (5.4%)	*χ* ^2^ = 3.47, *p* = 0.06	2/21 (9.5%)	17/240 (7.1%)	*χ* ^2^ = 0.17, *p* = 0.68
COVID first wave lockdown	19/78 (24.4%)	47/183 (25.7%)	*χ* ^2^ = 0.05, *p* = 0.82	23/75 (30.7%)	43/186 (23.1%)	*χ* ^2^ = 1.61, *p* = 0.20	4/21 (19%)	62/240 (25.8%)	*χ* ^2^ = 0.47, *p* = 0.49
COVID second wave lockdown	18/78 (23.1%)	59/183 (32.2%)	*χ* ^2^ = 2.2, *p* = 0.14	20/75 (26.7%)	57/186 (30.6%)	*χ* ^2^ = 0.40, *p* = 0.52	4/21 (19%)	73/240 (30.4%)	*χ* ^2^ = 1.20, *p* = 0.27
*χ* ^2^, *p*, Cramér's *V*	*χ* ^2^ = 10.6, *p* = 0.004, Cramér's *V* = 0.21	*χ* ^2^ = 34.7, *p* < 0.001, Cramér's *V* = 0.25	‐	*χ* ^2^ = 8.1, *p* = 0.017, Cramér's *V* = 0.20	*χ* ^2^ = 39.6, *p* < 0.001, Cramér's *V* = 0.27	‐	*χ* ^2^ = 0.95, *p* = 0.62	*χ* ^2^ = 44, *p* < 0.001, Cramér's *V* = 0.24	‐

**Figure 4:**
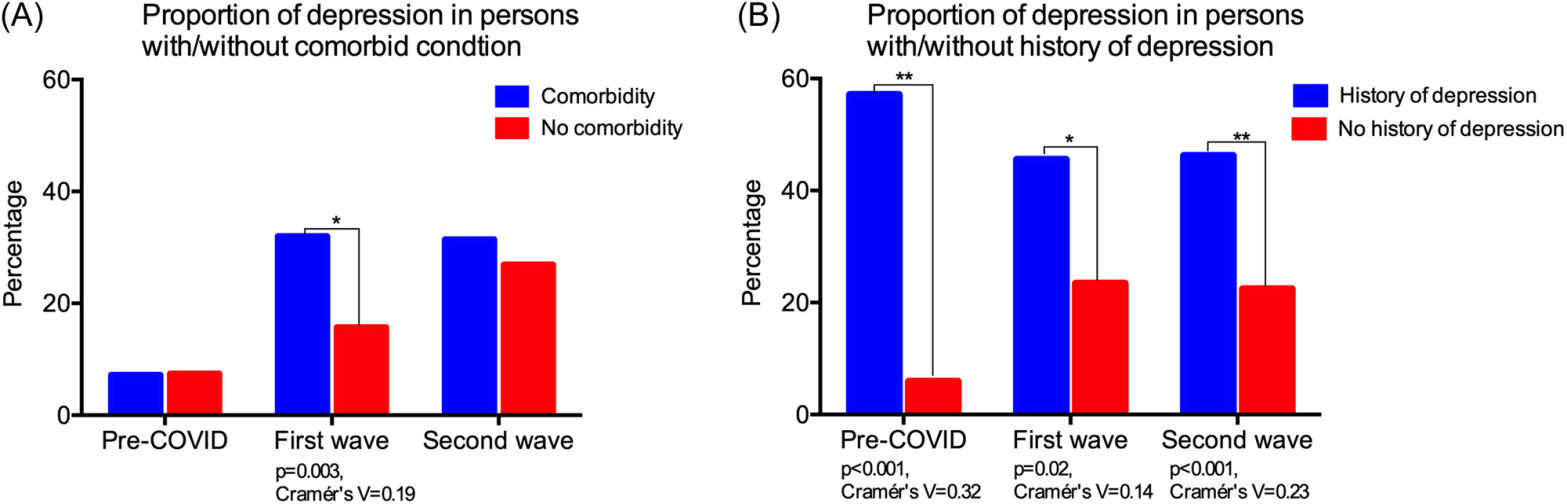
This figure highlights the differences in proportions of depression between subjects with/without comorbidities and with/without self‐reported history of depression among a longitudinal subgroup of rural cohort subjects (*n* = 261) who had depression assessments pre‐COVID as well as during the first wave and second waves of COVID.

Rural subjects with one or more comorbidities were found to have a significantly higher proportion of depression than those without comorbidities (32%; 49/153 vs. 15.7%; 17/108, *p* = 0.003) during the COVID first wave, as represented in Figure 4. However, no significant difference was observed between these two groups in the pre‐COVID and COVID second wave periods. The proportion of depression in subjects with past history (self‐reported) of depression was significantly higher than those without past history of depression during all three time periods (Figure 4); the difference was most prominent in the pre‐COVID period (57.1%; 4/7 vs. 5.9%; 15/254, *p* < 0.001) when compared to the COVID first wave (45.5%; 10/22 vs. 23.4%; 56/239, *p* = 0.023) and COVID second wave (46.2%; 36/78 vs. 22.4%; 41/183, *p* = 0.001) periods. We were unable to do a similar analysis in a longitudinal subgroup of subjects in the urban cohort due to low sample numbers.

## 4 DISCUSSION

This study highlights the importance of understanding the psychological impact of COVID‐19 in the aging, Indian population. To our best knowledge, this is the first study in India that has compared serially, proportions of depression during various stages of the pandemic in two diverse cohorts—rural and urban. We report significant variation in the trends of depression during these four periods (pre‐COVID, COVID first wave lockdown period, COVID inter‐wave period, and COVID second wave lockdown period) between these two cohorts. In the rural cohort, the proportion of subjects with depression rose from 8.3% (57/684) in the pre‐COVID period (2019) to 28.8% (211/733) during the first wave of COVID‐19. After the easing of lockdowns, that is, during the inter‐wave period, the proportion of depression dropped to 16.6% (76/458). Then, there was a sharp increase to 28.8% (176/611), once again, during the second wave of COVID‐19. This trend could imply that the rural subjects underwent considerable distress during first and second waves of the pandemic. However, potential reasons for this distress could be different during the first and second waves. The first wave of the pandemic is likely to have placed a substantial financial strain on this marginalized, rural population. The tight restrictions during the first wave significantly hampered the harvest, transport, and sale of mango produce in the Srinivaspura area, where majority of the population are mango farmers. This could have led to sizable financial burden, especially since these individuals hail from a low socioeconomic background and depend on agriculture for their livelihood. The significant drop in depression during the inter‐wave period is consistent with the substantial easing of restrictions, when some sense of normalcy returned, and the rural subjects were able to resume their work to a large extent. During the first wave of the pandemic, rural areas in India were much less affected in terms of the infection rates and case load. However, during the second wave, rural areas also had considerable spread of infection that was further compounded by shortage of adequate medical facilities to handle the crisis, which is the likely reason for the substantial increase in the depression, once again, during the second wave.

In the urban cohort, when compared to the proportion of pre‐COVID depression, there was no significant increase in depression during any time period after the onset of the pandemic. On the other hand, there was a statistically significant drop from 12% (38/317) in the pre‐COVID period to 6.1% (18/297) during the first wave. The reason for this is unclear, especially considering that urban Bangalore was one of the major hotspots of the pandemic in the state of Karnataka. Possible reasons include subjects who were severely depressed refrained from participating in the survey or had hesitancy expressing their distress or mental health problems during the telephonic survey. Subsequently, proportion of depression in urban subjects rose to 8.8% (18/204) in the inter‐wave period and then to 13.1% (40/305) during the second wave lockdown.

Proportions of subjects screened to have anxiety on GAD‐7 in both cohorts, during both waves of the pandemic were minimal. However, we did not have pre‐COVID anxiety assessments to make a comparison with data during COVID lockdown periods (we have now added this to our clinical assessment battery). However, it is interesting to note that the proportions of subjects with anxiety as compared to depression in both cohorts were substantially lower. One of the possible reasons could be that older adults in this part of the world could consider openly admitting or expressing anxiety or fear as a sign of weakness, whereas expressing distress or sadness could be more socially acceptable. It has also been previously shown that older persons are less likely to identify anxiety symptoms than depressive symptoms in themselves.^40^

On sub‐analyzing the longitudinal subgroup of 261 rural subjects who had undergone assessments in the pre‐COVID as well as COVID first and second waves, we found that subjects in the age group ≥65 years were significantly more depressed than those in the age group <65 years only during the first wave of the pandemic. Similarly, those subjects with one or more comorbidities were significantly more depressed than those without comorbidities only during the first wave. This could be explained by the fact that India had a very stringent lockdown during the first wave of COVID‐19, which grossly impacted access to routine healthcare services. This was probably more pronounced in the rural areas, where both healthcare infrastructure and access are very limited. Hence, it is likely that rural‐dwelling older adults ≥65 years and those with comorbidities faced severe difficulty in accessing healthcare for their medical problems, thus leading to higher depression. It is also possible that the older subjects (≥65 years) were more severely affected by the drastic restriction of social interactions owing to the strict lockdowns that accompanied the first wave. Prior studies in other parts of the world have clearly demonstrated the importance of social interaction for mental health^41^, ^42^ of older adults; social isolation has been associated with depression in elderly^43^ and is known to predict worse disease outcomes.^44^ On the other hand, the second wave's social restrictions in India were at a substantially lesser scale than the first wave's strictly enforced, nationwide lockdowns, which is potentially the reason why the trend discussed above was not seen during the second wave period.

Strengths of our study include comparison of depression and anxiety using the similar assessment tools in two aging populations with distinct population characteristics—(1) lower socioeconomic strata, less literate and marginalized rural subjects from the SANSCOG cohort and (2) middle/upper socioeconomic strata, highly educated and working‐class urban subjects from the TLSA cohort. Second, serially following up the same subgroup of rural subjects at three different time frames—pre‐COVID, COVID first wave, and second wave enabled us to get a clear picture of the changing proportions of depression as well as potential factors influencing this change. Limitations of our study include varying modes of administration and different versions of the depression assessment tool (in‐person assessments using GDS 30‐item version during the pre‐COVID period as compared to telephonic assessments using GDS 7‐item version during the COVID first and second wave lockdown periods). Also, we did not have adequate numbers to conduct sub‐analysis in the same subset of urban cohort subjects as we did for the rural subjects. Finally, these results may not be generalizable due to non‐probabilistic sampling method that was used in this study. We propose to conduct follow‐up assessments for depression and anxiety in our subjects in the coming months, with larger sample sizes, to further observe how trends in depression and anxiety play out in the future.

Geriatric depression is a mounting challenge in India, especially considering the sharp rise in its proportion of older persons in recent years.^45^ Wide variations have been reported in the prevalence of elderly depression in India depending on study settings, geographical regions, assessment tools used, and so on.^46^, ^47^ Depression in the elderly is known to be associated with higher risk for cognitive decline,^48^ physical disability,^49^, ^50^ frailty,^51^ and mortality.^52^ The current COVID‐19 crisis has further exposed the challenge of mental health disorders in the elderly and brought to light the challenges of delivering adequate mental healthcare to the elderly population of India.^53^ Prompt assessment of the impact of COVID‐19 on mental health of the elderly is essential to put in place mitigative measures or develop preventive strategies for the furture.^54^, ^55^ The treatment gap for mental health disorders in India is already very high (70%–92%)^14^ and the added burden of mental disorders related to COVID‐19 among rural elderly can present a very daunting challenge unless appropriate preventive public health measures are put in place. Considering challenges, such as stigma in developing countries like India, it is important to promote mental health awareness; another option would be to integrate mental health care in general/primary care services so that hesitation for visiting a psychiatrist is minimized. The District Mental Health Program launched in India is a good example for this, but the coverage needs to be expanded substantially. Further, in view of resource constraints, it is important to devise novel and cost‐effective strategies for large‐scale mental healthcare delivery, particularly during crisis situations. Telepsychiatry services could be one such option. A recent study from Italy has shown that virtual psychological interventions (telephonic/videoconferencing) during the COVID‐19 lockdown period were useful in the elderly, despite technological barriers.^56^ Our own experience of telephonically engaging with our rural (SANSCOG) cohort subjects during the first wave lockdown period to provide reassurance, awareness on COVID‐related safety precautions, and medical guidance, when necessary, was appreciated by our participants.^39^ However, it is important that these strategies are culturally acceptable and easily implementable through the existing primary healthcare infrastructure in India.

## AUTHOR CONTRIBUTIONS

**Jonas S. Sundarakumar**: Conceptualization; Investigation; Methodology; Project administration; Supervision; Validation; Visualization; Writing – original draft; Writing – review & editing. **Abhishek L. Menesgere**: Data curation; Formal analysis; Investigation; Methodology; Validation; Visualization; Writing – review & editing. **Shafeeq K. S. Hameed**: Data curation; Formal analysis; Investigation; Methodology; Supervision; Visualization. **Vijayalakshmi Ravindranath**: Conceptualization; Funding acquisition; Investigation; Methodology; Project administration; Resources; Supervision; Validation; Writing – review & editing.

## CONFLICT OF INTEREST

The authors declare no conflict of interest.

## ETHICS STATEMENT

This study involves human participants and was approved by the Centre for Brain Research—Institutional Ethics Committee (Ref: CBR/42/IEC/2021‐22).

## TRANSPARENCY STATEMENT

The lead author Jonas S. Sundarakumar affirms that this manuscript is an honest, accurate, and transparent account of the study being reported; that no important aspects of the study have been omitted; and that any discrepancies from the study as planned (and, if relevant, registered) have been explained.

## Supporting information

Supporting information.

Supporting information.

## Data Availability

Data will be shared in conformity with statutory requirements of the government of India, through the Alzheimer's Disease Data Initiative (ADDI) platform.
