# Supplementary material for: Depression and anxiety during the first and second waves of the COVID‐19 pandemic in two large, prospective, aging cohorts in rural and urban India
Source: Health Sci Rep. 2022 Nov 8;5(6):e901. doi: 10.1002/hsr2.901 (PMC9642812; doi:10.1002/hsr2.901)
Supplement: Supplementary file 1 — Supporting information. [file HSR2-5-0-s001.docx]

**Appendix I**

**Details of the rural and urban two cohort studies and their recruitment strategies**

The two cohort studies mentioned in this manuscript are both aimed at a comprehensive understanding of the diverse trajectories of aging, which in turn, will help in unravelling the risk and protective factors of aging-related, neurodegenerative disorders such as dementia. As it is known that the pathogenesis of dementia begins around 2 to 3 decades before diagnosable clinical symptoms appear^41^, in both cohorts, cognitively health adults aged 45 years and above are recruited to undergo detailed, multimodal (clinical, cognitive, biochemical, genetic and neuroimaging) evaluations along with periodic follow-up over several years^42^.

The rural study, namely, Srinivaspura Aging, Neurosenescence and COGnition (SANSCOG) study (projected n=10,000) is conducted in the villages of Srinivaspura, which is a sub-district (‘taluk’) in the district of Kolar in the state of Karnataka. On the other hand, the urban study, namely, Tata Longitudinal Study of Aging (TLSA) is conducted in the metropolitan city of Bangalore, the capital city of Karnataka. All assessments in both these studies are completely harmonized, thus rendering an unmatched opportunity to compare these two diverse, aging population groups. Further, clinical assessments in both studies include detailed neuropsychiatric assessments, including depression assessments using the Geriatric Depression Scale (GDS-30) that is performed by clinicians (registered medical practitioners or senior nurses), who undergo a 2-week intensive training program in administering the clinical scales digitally, using handheld digital devices.

**Recruitment Strategy**: Rural participants are recruited, through geographical cluster sampling strategy from Srinvaspura ‘taluk’ (sub-district). In India, the primary units of the public-funded healthcare system are Primary Health Centres (PHCs). Each PHC caters to the primary healthcare needs of around 20,000 to 30,000 people located in its surrounding villages. Srinivaspura taluk has a total of 13 PHCs, out of which, we randomly selected 6 PHCs for SANSCOG study’s recruitment. Our field team works in collaboration with local public health officials as well local community health workers, called ASHAs (Accredited Social Health Activists), to systematically recruit eligible and consenting individuals from the villages attached to each PHC. Awareness programs are conducted in each village, in addition to public announcements and distribution of flyers. On the other hand, the urban subjects are recruited, through convenience sampling, from urban areas in Bangalore city. Study awareness is created through newspaper advertisements, distribution of flyers, social media as well as by conducting awareness programs in large apartment complexes / gated communities.

At the time of preparing this manuscript, n=2368 rural subjects and n=483 subjects had been recruited in the respective cohorts and had completed their baseline assessments.
