# Supplementary material for: Depression and anxiety during the first and second waves of the COVID‐19 pandemic in two large, prospective, aging cohorts in rural and urban India
Source: Health Sci Rep. 2022 Nov 8;5(6):e901. doi: 10.1002/hsr2.901 (PMC9642812; doi:10.1002/hsr2.901)
Supplement: Supplementary file 2 — Supporting information. [file HSR2-5-0-s002.docx]

**Appendix II**

**Instruments used for telephonic assessment of depression and anxiety**

**Geriatric Depression Scale (GDS-7)**

This is a validated, self-reported, screening tool for depression that consists of 7 ‘yes’ or ‘no’ questions. Questions answered ‘yes’ are scored with 1 point and those answered ‘no’ are scored zero. The total score is calculated by adding the scores of all the 7 answers. A cut-off of ≥ 2 has a sensitivity of 93% and specificity of 91% to detect depression.

**Generalized Anxiety Disorder Questionnaire (GAD-7)**

This is a self-reported screening tool that has been well-validated to detect Generalized Anxiety Disorder (GAD) as well as other common anxiety disorders, such as panic disorder, social anxiety disorder and post-traumatic stress disorder (PTSD). There are 7 questions, whose responses are indicative of the frequency of the symptom in question – categorized as 0 – 'not at all', 1 – 'several days', 2 – 'more than half the days' and 3 – 'nearly every day. The total score is calculated by adding the scores of all the 7 responses. A cut-off score of 10 or more has been found to be optimal in detecting GAD with a sensitivity of 89% and specificity of 82%. Further, higher scores are indicative of greater severity of anxiety.

In the aging, rural cohort, which had poor literacy and low familiarity with use of gadgets such as mobile phones, during the first wave lockdown period, we initially piloted the telephonic assessments in 417 subjects with a single trained assessor administering the questionnaires telephonically to each participant. However, since the inter-rater reliability between assessors was found to be unsatisfactory (owing to difficulty in the assessor presenting the questions / interpreting their responses in a culturally appropriate manner), we subsequently implemented a rigorous ‘panel method’ for the telephonic assessments. Panels comprising of three assessors each were formed. After detailed discussions with field experts and locally-residing project staff, a Standard Operating Procedure (SOP) including a translated script (in the local language and using carefully chosen words in the local dialect) was created and shared with each panel. The assessors were trained and underwent mock sessions to ensure uniformity in administration of the questions and marking of the responses. Subsequently, each participant was interviewed by a panel of 3 assessors, wherein one (primary) assessor administered the questions and the responses provided by the subjects were independently rated by the primary as well as the two other (secondary) assessors. After the interview, the panel compared and discussed the ratings of all three assessors and a common consensus was reached. In case there was inability to reach a consensus , we had decided to exclude the data from analysis; however, we did not encounter any such cases. This method provided excellent inter-rater reliability between the assessors. This study includes only the assessments that were done using this panel method and the 427 pilot assessments have been excluded (Figure 1). Assessments in the rural cohort during the second wave lockdown period were conducted exclusively using the panel method. The above method was not necessary in the urban subjects since they were highly educated, and a majority were already familiar with such remote methods of undertaking surveys.
